# The epidemiology of dying within 48 hours of presentation to emergency departments: a retrospective cohort study of older people across Australia and New Zealand

**DOI:** 10.1093/ageing/afae067

**Published:** 2024-04-09

**Authors:** Amy L Sweeny, Nemat Alsaba, Laurie Grealish, Kerina Denny, Bill Lukin, Andrew Broadbent, Ya-Ling Huang, Jamie Ranse, Kristen Ranse, Katya May, Julia Crilly

**Affiliations:** Department of Emergency Medicine, Gold Coast Hospital and Health Service, Gold Coast University Hospital, Gold Coast, Queensland, Australia; Menzies Health Institute Queensland, Griffith University, Gold Coast, Queensland, Australia; Faculty of Health Sciences and Medicine, Bond University, Gold Coast, Queensland, Australia; Department of Emergency Medicine, Gold Coast Hospital and Health Service, Gold Coast University Hospital, Gold Coast, Queensland, Australia; Faculty of Health Sciences and Medicine, Bond University, Gold Coast, Queensland, Australia; Menzies Health Institute Queensland, Griffith University, Gold Coast, Queensland, Australia; Nursing & Midwifery Education & Research Unit, Gold Coast Hospital and Health Service, Gold Coast, Queensland, Australia; Department of Emergency Medicine, Gold Coast Hospital and Health Service, Gold Coast University Hospital, Gold Coast, Queensland, Australia; Department of Intensive Care Medicine, Gold Coast Hospital and Health Service, Gold Coast, Queensland, Australia; Faculty of Health and Behavioural Sciences, School of Medicine, University of Queensland, Brisbane, Queensland, Australia; Department of Emergency Medicine, Royal Brisbane and Women’s Hospital, Brisbane, Queensland, Australia; Faculty of Health Sciences and Medicine, Bond University, Gold Coast, Queensland, Australia; Supportive and Specialist Palliative Care, Gold Coast Hospital and Health Service, Gold Coast, Queensland, Australia; Department of Emergency Medicine, Gold Coast Hospital and Health Service, Gold Coast University Hospital, Gold Coast, Queensland, Australia; Faculty of Health (Nursing), Southern Cross University, Gold Coast, Queensland, Australia; School of Nursing and Midwifery, Griffith University, Gold Coast, Queensland, Australia; Department of Emergency Medicine, Gold Coast Hospital and Health Service, Gold Coast University Hospital, Gold Coast, Queensland, Australia; Menzies Health Institute Queensland, Griffith University, Gold Coast, Queensland, Australia; School of Nursing and Midwifery, Griffith University, Gold Coast, Queensland, Australia; Menzies Health Institute Queensland, Griffith University, Gold Coast, Queensland, Australia; School of Nursing and Midwifery, Griffith University, Gold Coast, Queensland, Australia; Department of Emergency Medicine, Gold Coast Hospital and Health Service, Gold Coast University Hospital, Gold Coast, Queensland, Australia; School of Nursing and Midwifery, Griffith University, Gold Coast, Queensland, Australia; Department of Emergency Medicine, Gold Coast Hospital and Health Service, Gold Coast University Hospital, Gold Coast, Queensland, Australia; Menzies Health Institute Queensland, Griffith University, Gold Coast, Queensland, Australia; School of Nursing and Midwifery, Griffith University, Gold Coast, Queensland, Australia

**Keywords:** aged, emergency care, palliative care, end-of-life care, epidemiology, older people

## Abstract

**Background:**

Emergency department (ED) clinicians are more frequently providing care, including end-of-life care, to older people.

**Objectives:**

To estimate the need for ED end-of-life care for people aged ≥65 years, describe characteristics of those dying within 48 hours of ED presentation and compare those dying in ED with those dying elsewhere.

**Methods:**

We conducted a retrospective cohort study analysing data from 177 hospitals in Australia and New Zealand. Data on older people presenting to ED from January to December 2018, and those who died within 48 hours of ED presentation, were analysed using simple descriptive statistics and univariate logistic regression.

**Results:**

From participating hospitals in Australia or New Zealand, 10,921 deaths in older people occurred. The 48-hour mortality rate was 6.43 per 1,000 ED presentations (95% confidence interval: 6.31–6.56). Just over a quarter (n = 3,067, 28.1%) died in ED. About one-quarter of the cohort (n = 2,887, 26.4%) was triaged into less urgent triage categories. Factors with an increased risk of dying in ED included age 65–74 years, ambulance arrival, most urgent triage categories, principal diagnosis of circulatory system disorder, and not identifying as an Aboriginal or Torres Strait Islander person. Of the 7,677 older people admitted, half (n = 3,836, 50.0%) had an encounter for palliative care prior to, or during, this presentation.

**Conclusions:**

Our findings provide insight into the challenges of recognising the dying older patient and differentiating those appropriate for end-of-life care. We support recommendations for national advanced care planning registers and suggest a review of triage systems with an older person-focused lens.

## Key Points

- In Australia and New Zealand, the 48-hour mortality rate for older persons was 6.43 per 1,000 emergency presentations.
- About half of the older people admitted to hospital had a prior palliative-type presentation or admission in the last 6 months.
- Factors associated with an increased risk of dying in the emergency department included arrival by ambulance and a triage category of 1, 2 or 5.

## Introduction

Care of the older person in the emergency department (ED) is a priority area for emergency care research from the perspectives of emergency physicians in the UK [1] and Australia [2, 3], and ED patients [4]. The need to prioritise the care of older person is underpinned by several challenges including an ageing population (which is evident in every country in the world) [5] and over-representation of older people in the ED [6]. For example, in Australia, people aged ≥65 years accounted for ~21% of ED presentations but only 16% of the population [7]. With ageing comes typically more medical problems, polypharmacy, and more functional and cognitive challenges, which may contribute to the ED presentation and influence outcomes [8].

To support care of the older person in the ED, some research priorities consider improvements around medication management and compliance, falls screening and interventions, better screening for substance use disorders, and activities of daily living screening before discharge, including amongst older persons with functional decline [9, 10]. These priorities focus on improving quality of life and preventing functional decline; research focussed on end-of-life care delivered within the ED is often overlooked.

Historically, the ED has been designed to manage ‘traditional’ emergencies (e.g. trauma), and acute emergencies (e.g. heart attacks and strokes). However, with the ageing population and increased life expectancy, EDs are experiencing an increase in people presenting to ED with complications associated with advanced, and often multiple, chronic diseases. ED clinicians are increasingly recognising the need for and upskilling themselves to manage an ageing population [11], whose optimal care may require end-of-life care in certain circumstances.

***End-of-life care** is a form of palliative care ‘that can be provided to people who may be expected to die within the next few hours, days, or months’* [12]*. People at end of life include those whose death is imminent, whether due to an advanced incurable condition or a sudden catastrophic event* [12]*.*

***Palliative care** uses a holistic approach to support comfort measures for people with a life-limiting illness* [12]*. It offers a support system to help patients live as actively as possible until death* [13]*. Specialist palliative care is one component of palliative care service delivery* [13]*.*

*In this manuscript, we define end of life as any death occurring within 48 hours of ED presentation, regardless of cause and/or reversibility of presentation reasons.*

A visit to the ED proximal to death is not unusual. One US study found that about one-tenth of people (all ages) experience their death in the ED, and approximately one-third of people who died had been seen in ED within the month before their death [14]. The ED death rate for patients aged ≥60 years was 8.0 per 1,000, with 1 in 12 persons aged ≥80 years dying in ED or within the month after their visit [14]. Few other estimates of the incidence of death in ED or shortly thereafter exist. One population-based Korean study reports an ED death rate of 0.3% (all ages), and that approximately one-quarter of patients who died in ED would have been considered appropriate for palliative care based on pre-existing health conditions [15]. A second single-site study from Singapore found that more than half of older persons who died in ED were chronically unwell and more likely to benefit from palliative care [16]. Such epidemiological data are lacking for Australia and New Zealand, and the generalisability of previous studies is limited by differences in healthcare models and provision.

Although a common place to die, whether the ED is a preferred or suitable place for death according to patients at end of life has not been explored. In one study, symptom management occurred less frequently in ED compared to the wards for people at end of life who had symptoms of dyspnoea, anxiety and/or agitation [17]. Moreover, staff caring for dying patients concede that death in the ED is not optimal for patients receiving palliative care [18], and more than half of patients at end of life report wanting to die at home [19–21], although this varies by symptom severity and family situation [22]. For people aged >75 years, a recent French study found that spending the hours from midnight to 08:00 in ED carried increased risks of nosocomial infection, falls and in-hospital mortality, especially for those with decreased autonomy [23]. Furthermore, since patients’ preferences at end of life include having loved ones around [22, 24, 25] many EDs may not be able to accommodate the space and privacy required. ED clinicians themselves can also face significant uncertainties, challenges and frustrations in the management of patients near their expected end of life [26, 27]. Hence, further describing patients that die within ED compared to elsewhere (at home or in hospital) can refine our understanding of the need for end-of-life care provision within EDs.

Understanding the epidemiology of people presenting at end of life is also key to informing policy and management decisions about resource allocation within the ED and other parts of the health system, yet estimates of the need for end-of-life care provision in EDs are scarce [28]. This study provides an estimate pertinent to the Australian and New Zealand health care setting, and describes the cohort of patients imminently dying who present to ED, along with factors associated with death within the ED itself.

## Methods

### Study design

A retrospective cohort study using de-identified data was conducted to describe patients who present to Australian and New Zealand EDs and die within the subsequent 48 hours, and to quantify the incidence rate of imminent death in these jurisdictions. Older patients that died in ED are compared to those that died elsewhere.

### Setting

Data pertaining to all EDs participating in the Health Roundtable Project (https://home.healthroundtable.org) were requested. Health Roundtable is a health performance improvement not-for-profit organisation that maintains a collection of linked ED and inpatient data from participating public hospitals across Australia and New Zealand. At the time of this study, 177 EDs were submitting data to Health Roundtable.

### Population

Our inclusion criteria were age ≥65 years, ED presentation between 1 January 2018 and 31 December 2018, and death during an episode continuous with the ED presentation (regardless of location of death), within 48 hours of ED presentation. Patients who were dead upon ED arrival were excluded. Deaths that occurred outside the hospital setting (after the ED episode of care) were included if the patient was under hospice or hospital-in-the-home care, or if the hospital became aware of the death following discharge. Some participating EDs did not have any deaths of older people within 48 hours; the ED presentation denominators were still included in all calculations.

### Data collection

Variables provided by Health Roundtable included de-identified facility information, ED data (e.g. Australasian Triage Scale category (ATS), mode of arrival, principal diagnosis, ED arrival date/time, ED departure date/time and destination) and inpatient data when applicable (e.g. admission date/time, discharge date/time, principal and secondary diagnoses (using International Classification of Diseases, Tenth Revision (ICD-10) codes), intensive care unit (ICU) admission and diagnostic related group). In some cases, inpatient data included changes in care type (e.g. from active management to palliative care management). The ATS is a clinical tool used by appropriately trained and experienced staff members to prioritise patients presenting to ED according to their clinical urgency. The ATS has five categories from category 1, warranting an immediate response, to category 5 (assessment and treatments to start within 120 minutes) [29].

Health Roundtable also derived several variables specifically for this project. These included ICU hours, ventilation hours and a four-option discharge destination variable for those that did not die in ED that grouped ICU, coronary care unit and high-dependency unit into one discharge category; medical and surgical wards into another discharge category; and short-stay wards grouped into a third and fourth discharge category, depending on whether managed by ED staff or other staff. A palliative care indicator was derived by Health Roundtable, defined as any diagnosis of ICD-10 code Z51.5 (Encounter for palliative care) occurring for an individual during any presentation or admission that occurred during the prior 180 days. Additional files were supplied of those variables with multiple recurring entries, e.g. final presentations’ secondary diagnoses and secondary procedures, and details of prior ED and hospital visits to the same hospital in the 180 days prior (e.g. ED length of stay (LOS), inpatient and/or ICU LOS, principal diagnosis, care type).

### Statistical analysis

The number of deaths within 48 hours was tallied within 5-year age groups, and age-group-specific death rates per 1,000 presentations were calculated using the Health Roundtable denominators supplied. A 95% confidence interval (CI) for each death rate was calculated using the Wilson Score method and publicly available statistical software (https://www.openepi.com/Menu/OE_Menu.htm). Additional descriptive and inferential statistical analyses were performed using Python 3.7. Patient age and LOS variables were assessed for normality; means or medians were calculated as appropriate, along with standard deviations or interquartile ranges (IQRs). For patients that were admitted, additional information on ICU LOS, ventilator hours, procedures and principal and secondary diagnoses was summarised. Categorical data were further summarised by location of death (ED or other ward, ICU, home or short-stay unit). Where statistical tests were appropriate, the chi-square test or chi-square test for trend was used to compare distributions for categorical variables. Risk ratios (RRs) and 95% CIs were calculated by performing univariate logistic regression modelling, one variable at a time, with the outcome of interest being death in ED. Confidence intervals that did not cross over 1.0 and differences with a *P*-value of <0.05 were considered statistically significant. Reference categories used were based on previous research, cell size or the most logical group to support interpretation. For example, ATS 3 was selected as the reference category as it is the most common triage category assigned in the general population of ED presenters [7]. The major diagnostic category (MDC) of ‘nonspecific symptoms and signs’ was used as the reference for diagnosis as it was a top 5 MDC and it was logical to compare this undifferentiated group to those with a clear diagnosis. Multivariate modelling was not performed.

### Ethical considerations

The study received Human Research Ethics Committee approval from the Health Service (HREC/2019/QGC/53211) and Griffith University (2020/529).

## Results

### Sample characteristics and incidence of death within 48 hours of ED presentation

Of the 177 participating hospitals, 159 reported one or more death of an older person in the 48-hour period.

**Figure 1:**
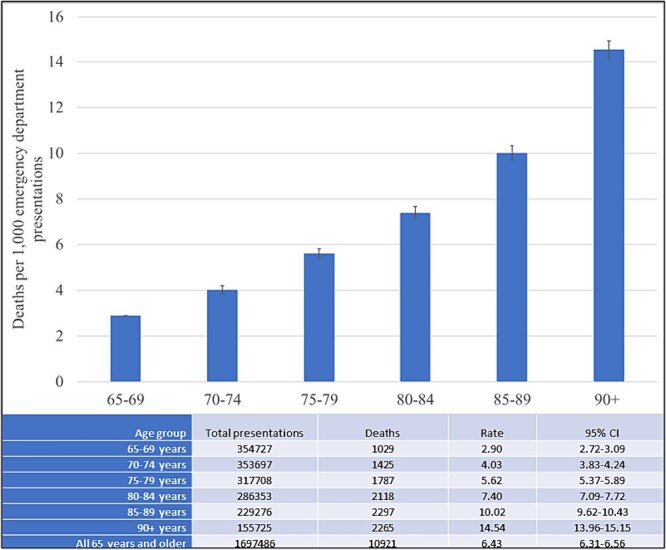
Deaths within 48 hours of emergency department presentation for older people in Australia and New Zealand.

Figure 1 outlines the incidence of death within 48 hours of ED presentation across this cohort. The overall 48-hour mortality rate for older persons presenting to EDs was 6.43 per 1,000 ED presentations (95% CI: 6.31–6.56). The rate increased with increasing 5-year age group, reaching 10.0 per 1,000 for octogenarians and 14.5 per 1,000 for nonagenarians.

### Demographics overall and by place of death, and ED length of stay

Table 1 describes key demographics of the cohort of older individuals who died within 48 hours of ED presentation, along with demographic differences between those who died in ED compared to elsewhere. Australian presentations constituted 78.4% of the cohort and Aboriginal and/or Torres Strait Islander people represented 1.7% of the Australian cohort. Māori people represented 11.1% of the New Zealand cohort.

**Table 1:** Demographic characteristics of older persons (i.e. ≥65 years) dying within 48 hours of ED triage

	All persons (n = 10,921)^a^		Persons died in ED (n = 3,067, 28.5%)		Persons died after admitted^b^ (n = 7,677, 71.5%)		*P*-value	Univariate risk ratio (95% CI) for dying in ED
**Age: median (IQR)**	**82 (75–88)**		**82 (75–88)**		**83 (76–89)**		<0.001^c^	Not applicable
**Characteristic**	**n**	**%**	**n**	**%**	**n**	**%**	***P*-value**	**Univariate risk ratio (95% CI) for dying in ED**
**Sex**								
Male	5,637	51.6	1,618	29.2	3,926	70.8	0.128	1.0 (1.0–1.0)
Female	5,282	48.4	1,448	27.9	3,750	72.1		1.0 (reference)
**Age group (years) (65–118)**							<0.001^d^	
65–69 years	1,029	9.4	312	30.9	698	69.1		1.3 (1.1–1.5)
70–74 years	1,425	13.0	444	31.6	961	68.4		1.3 (1.1–1.5)
75–79 years	1787	16.4	522	29.8	1,229	70.2		1.2 (1.0–1.4)
80–84 years	2,118	19.4	577	27.7	1,506	72.3		1.1 (0.9–1.2)
85–89 years	2,297	21.0	629	27.8	1,632	72.2		1.1 (1.0–1.2)
90+ years	2,265	20.7	583	26.1	1,651	73.9		1.0 (reference)
**Jurisdiction**							0.905	
Australia	8,558	78.4	2,409	28.5	6,038	71.5		1.0 (0.9–1.1)
New Zealand	2,363	21.6	658	28.6	1,639	71.4		1.0 (reference)
**Australian state**							<0.001	
QLD	1960	22.9	477	24.8	1,446	75.2		0.8 (0.7–0.9)
NSW	2,324	27.2	645	27.9	1,666	72.1		1.0 (0.8–1.1)
TAS	293	3.4	60	34.3	115	65.7		1.3 (0.9–1.8)
WA	716	8.4	323	46.1	377	53.9		2.1 (1.8–2.5)
VIC	2,424	28.3	164	24.6	502	75.4		1.0 (reference)
SA	666	7.8	698	28.8	1723	71.2		0.8 (0.7–1.0)
Other Australia	175	2.0	42	16.7	209	83.3		0.5 (0.4–0.7)
**Indigenous status (Australian only)**							0.004	
Any Aboriginal or Torres Strait Islander	125	1.5	21	16.9	103	83.1		0.9 (0.8–0.9)
Neither or not stated	8,433	98.5	2,388	28.7	5,935	71.3		1.0 (reference)
**Ethnicity (New Zealand only)**							0.118	
Māori	266	11.3	61	25.1	182	74.9		0.9 (0.9–1.0)
Other	2053	86.9	589	29.3	1,421	70.7		1.0 (reference)
Missing or not stated	44	1.9	8	18.2	36	81.8		Not included

^a^Includes 177 patients for whom place of death was not able to be determined.

^b^Admission also includes deaths occurring in short-stay units.

^c^Mann–Whitney *U* test.

^d^Chi-square for trend test.

About one-quarter of the cohort (n = 3,067, 28.1%) died in the ED, and 7,677 died following inpatient admission (70.3%). The place of death was indeterminate for 177 (1.6%). The likelihood of dying in ED decreased with increasing age category. Aboriginal and Torres Strait Islander people were less likely to die in ED; RR (95% CI) = 0.9 (0.8–0.9).

### Presentation and clinical characteristics overall

Table 2 reports the presentation and clinical characteristics of the cohort overall and by location of death (ED versus elsewhere). For each participating hospital with at least one death during the year (n = 159), the number of deaths ranged from 1 to 277 deaths (median: 50; IQR: 20–90). Principal referral centres accounted for 4,724 (43.3%) of all deaths. About 1 in 8 patients (14.1%) presented between midnight and 06:00; 1 in 10 arrived by their own transport, and most self-referred (n = 7,920, 72.5%), rather than being referred in by a general practitioner (Table 2). About one-quarter of the cohort (n = 2,887, 26.4%) was triaged into the less urgent categories (ATS 3, 4 or 5). The median time spent in ED for older persons prior to death in ED or transfer to another area was 4.7 hours (IQR: 3.0–7.3); this was similar whether the patient was admitted or died in ED. The range of ED LOS for the cohort was 15 minutes to 20 hours for those who died in ED and 15 minutes to 43 hours for those admitted.

**Table 2:** Presentation and clinical characteristics of patients who died in the emergency department (ED) versus elsewhere

	**All persons** ^**a**^ **(n = 10,921)**		**Persons died in ED (n = 3,067)**		**Persons admitted (n = 7,677)**		***P*-value**	**Univariate risk ratio (95% CI) for dying in ED**
	**n**	**%**	**n**	**%**	**n**	**%**		
**Type of hospital**							<0.001^b^	
Principal referral	4,724	43.3	1,482	31.7	3,186	68.3		1.0 (reference)
Public acute group A hospitals	3,840	35.2	1,032	27.3	2,747	72.7		0.8 (0.7–0.9)
Public acute group B hospitals	1793	16.4	466	26.4	1,301	73.6		0.8 (0.7–0.9)
Public acute group C hospitals, women’s hospitals	481	4.4	81	18.1	366	81.9		0.5 (0.4–0.6)
Public acute group D hospitals	83	0.8	6	7.2	77	92.8		0.2 (0.1–0.4)
**Time of triage/presentation**							<0.001	
06:00–11:59	3,151	28.9	847	27.6	2,255	29.4		0.9 (0.8–1.0)
12:00–17:59	3,508	32.1	901	29.4	2,546	33.2		0.8 (0.7–0.9)
18:00–23:59	2,724	24.9	858	28.0	1819	23.7		1.1 (0.9–1.2)
00:00–05:59	1,538	14.1	461	15.0	1,057	13.8		1.0 (reference)
**Mode of arrival**							<0.001	
Self-presented	1,104	10.1	238	7.8	851	11.1		1.0 (reference)
Ambulance	9,542	87.4	2,738	89.3	6,668	86.9		1.5 (1.3–1.7)
Not stated/unknown	275	2.5	91	3.0	158	2.1		2.1 (1.5–2.8)
**Referral source**							<0.001	
Self	7,920	72.5	2,445	79.7	5,394	70.3		1.0 (reference)
General/primary or other health practitioner	504	4.6	83	2.7	414	5.4		0.4 (0.3–0.6)
Transfer-in	852	7.8	187	6.1	629	8.2		0.7 (0.6–0.8)
Other	869	8.0	200	6.5	660	8.6		0.7 (0.6–0.8)
Not collected	776	7.1	152	5.0	580	7.6		0.6 (0.5–0.7)
**Triage category**							<0.001^b^	
1	3,634	33.3	1,670	54.5	1911	24.9		5.0 (4.4–5.7)
2	4,395	40.2	982	32.0	3,353	43.7		1.7 (1.5–1.9)
3	2,430	22.3	353	11.5	2026	26.4		1.0 (reference)
4	428	3.9	49	1.6	368	4.8		0.8 (0.6–1.1)
5	29	0.3	11	0.4	17	0.2		3.7 (1.7–8.0)
Missing	5	0.0	2	0.1	3	0.0		Not included
**Time of ED discharge**							<0.001	
06:00–11:59	2,473	22.6	643	21.0	1800	23.4		1.0 (reference)
12:00–17:59	2,954	27.0	898	29.3	2011	26.2		1.3 (1.1–1.4)
18:00–23:59	2,969	27.2	855	27.9	2048	26.7		1.2 (1.0–1.3)
00:00–05:59	2,525	23.1	671	21.9	1818	23.7		1.0 (0.9–1.2)
**Length of stay**								
ED length of stay (hours), median [IQR]	**4.7 [3.0–7.3]**		**4.7 [2.9–7.2]**		**4.7 [3.0–7.4]**			
Total length of stay (hours), median [IQR]	**16.0 [7.0–29.5]**		**4.7 [2.9–7.2]**		**23.0 [13.1–34.0]**			
	**n**	**%**	**n**	**%**	**n**	**%**		
**ED diagnosis (MDC)** ^**c**^								
Circulatory system	3,117	28.5	1,237	40.3	1833	23.9	<0.001	1.2 (1.1–1.3)
Respiratory system	1,674	15.3	387	12.6	1,264	16.5	<0.001	0.7 (0.6–0.8)
Nonspecific symptoms and signs	1,508	13.8	493	16.1	1,001	13.0		1.0 (reference)
Infectious and parasitic	733	6.7	183	6.0	541	7.0	<0.001	0.8 (0.7–0.9)
Digestive system	572	5.2	112	3.7	453	5.9	<0.001	0.6 (0.5–0.7)
Injury, poisoning, external causes	529	4.8	112	3.7	412	5.4	<0.001	0.6 (0.5–0.8)
Health services/other	389	3.6	59	1.9	317	4.1		
Neoplasms	329	3.0	66	2.2	254	3.3		
Genitourinary	263	2.4	39	1.3	221	2.9		
Endocrine	151	1.4	26	0.8	124	1.6		
Mental health	85	0.8	13	0.4	71	0.9		
Missing	1,571	14.4	340	11.1	1,186	15.4		
**Commonly occurring diagnoses (>90 instances)**							Not calculated	
Cardiac arrest	886	8.1	647	21.1	224	2.9		
Stroke or subarachnoid haemorrhage	773	7.1	215	7.0	550	7.2		
Sepsis	686	6.3	177	5.8	501	6.5		
Influenza and pneumonia	593	5.4	127	4.1	460	6.0		
Chest pain or acute coronary syndrome	446	4.1	123	4.0	318	4.1		
COPD or emphysema	312	2.9	62	2.0	244	3.2		
Dyspnoea signs	270	2.5	68	2.2	201	2.6		
Heart failure	268	2.5	56	1.8	209	2.7		
Aspiration pneumonia	248	2.3	77	2.5	170	2.2		
Respiratory failure	207	1.9	89	2.9	114	1.5		
Traumatic head injury	199	1.8	51	1.7	147	1.9		
Death, sudden not otherwise specified	180	1.6	173	5.6	4	0.1		
Palliative care	168	1.5	19	0.6	142	1.8		
Gastrointestinal bleed	159	1.5	37	1.2	118	1.5		
Lower respiratory tract infection	151	1.4	28	0.9	121	1.6		
Bowel obstruction	150	1.4	28	0.9	121	1.6		
Acute renal failure	140	1.3	15	0.5	123	1.6		
Abdominal pain not otherwise specified	139	1.3	22	0.7	117	1.5		
Somnolence, stupor or coma	115	1.1	31	1.0	81	1.1		
Acute pulmonary oedema	107	1.0	28	0.9	77	1.0		
Hip fracture	97	0.9	8	0.3	88	1.1		
Urinary tract infection	93	0.9	20	0.7	73	1.0		

Risk ratios were calculated for only the most common major diagnostic categories were considered (top 5).

CI, confidence interval; GP, general practitioner; SAH, subarachnoid haemorrhage; COPD, chronic obstructive pulmonary disease.

^a^Includes 177 patients who were not assignable to a place of death (ED or elsewhere).

^b^Chi-square for trend test.

^c^MDC: major diagnostic category.

The most common diagnoses on ED discharge were cardiac arrest, stroke, sepsis and influenza/pneumonia, accounting for 2,938 (31.4%) of the 9,350 older people with diagnoses recorded. People diagnosed with neoplasms (n = 329, 3.0%) or injury/poisoning/external causes (n = 529, 4.8%) comprised a relatively small proportion of deaths overall, including those in ED.

### Presentation and clinical characteristics by place of death

Considering location of death, death within the ED became less likely as facility size decreased (chi-square for trend *P* < 0.001) (Table 2). Death in ED was more likely for patients triaged into categories of 1, 2 or 5 compared to triage category 3 presentations and for patients who arrived by ambulance (Table 2). Presentation to ED between the hours of 12:00 and 17:59 conferred a decreased risk of dying in ED; RR (95% CI) = 0.8 (0.7–0.9). However, those who died between the hours of 12:00 and 23:59 (noon-midnight) were more likely to die in ED.

Regarding clinical diagnoses, older people presenting due to circulatory system disorders were more likely to die in the ED compared to the undifferentiated group of patients presenting with nonspecific symptoms or signs; RR (95% CI) = 1.2 (1.1–1.3). Circulatory system disorders were disproportionately represented amongst patients that died in ED; 40.3% of the 3,070 older people with this primary diagnosis died within the ED. Nearly three-quarters of older persons with a cardiac arrest diagnosis who died within 48 hours died in the ED (n = 647 of 886 (73.0%)).

### Care delivery characteristics, consultations and additional diagnoses for patients admitted

Table 3 shows additional care delivery characteristics, consultations and diagnoses for the 7,677 older persons admitted from ED who died within 48 hours of presentation.

**Table 3:** Care delivery characteristics, consultations and additional diagnoses for 7,677 older persons admitted from ED and dying within 48 hours of ED presentation

**Characteristic**	n	%
**ED short stay first**		
Yes	317	4.1
No	5,338	69.5
Missing	2022	26.3
**Admission type**		
Emergency	7,109	92.6
Planned	57	0.7
Not assigned/unknown	511	6.7
**ICU care received**		
Yes	1,193	15.5
No	6,484	84.5
**ICU hours, n = 1,193, median [IQR]**	**15 [7–24]**	
**Ventilation required**		
Yes	651	8.5
No	7,026	91.5
**Ventilation hours, n = 651, median [IQR]**	**14 [6–24]**	
**Principal procedures with >20 instances**		
Allied health consult	979	12.8
Non-invasive ventilation support	733	9.5
Invasive ventilation support	543	7.1
Social work consult	530	6.9
Transfusion of blood and products	307	4
Coronary angiography with or without stenting	147	1.9
Surgery, hip	42	0.5
Surgery, incision musculoskeletal	35	0.5
Loading of delivery device, medications	25	0.3
Procedure code listed, not included above	494	6.4
**Top 10 AR-DRG groups**		
Respiratory infections	1,004	13.1
Sepsis	870	11.3
Stroke	863	11.2
Heart failure	393	5.1
ACS, no intervention	365	4.8
Neoplasm	299	3.9
COPD	291	3.8
**Chronic conditions contributing (principal or secondary diagnosis)**		
Diabetes	5,460	71.1
Cancer	4,209	54.8
Arthropathies	2,469	32.2
Heart failure	1,920	25
Lung disease (COPD)	1,934	25.2
Digestive system disorders, NOS	1,644	21.4
Renal disease	1,525	19.9
Hypertension	1,442	18.8
Atrial fibrillation	1,174	15.3
Cognitive impairment (chronic)	1,157	15.1
**Care type**		
Acute	6,986	91
Palliative	683	8.9
Other, not listed	8	0.1
**Palliative care encounter** ^**a**^		
Yes, pre-existing	3,279	42.7
Yes, after admission	557	7.3
No, neither	3,841	50
**Time of discharge (death)**		
06:00–11:59	1,800	23.4
12:00–17:59	2,011	26.2
18:00–23:59	2,048	26.7
00:00–05:59	1,818	23.7

ICU, intensive care unit; AR-DRG, Australian-Refined diagnostic related group; COPD, chronic obstructive pulmonary disease; ACS, acute coronary syndrome; NOS, not otherwise specified.

^a^Received a diagnosis of palliative care encounter (ICD-10 Z51.5) during a prior presentation/admission in the last 180 days (pre-existing) or during this admission.

Overall, around 16% of the cohort (n = 1,193) were admitted to the ICU and 8.5% (n = 651) had between 6 and 24 hours of ventilation before they died. The most common procedures (>20 instances) received by patients included non-invasive ventilation support (9.5%, n = 733), invasive ventilation support (7%, n = 543), blood transfusions (4%, n = 307) and coronary angiography with or without stenting (2%, n = 147). The most common consultations requested were allied health (13%, n = 979) and social work (7%, n = 530).

Final discharge diagnoses most frequently reported included respiratory infections (13%, n = 1,004), sepsis (11%, n = 870), stroke (11%, n = 863), heart failure (5%, n = 393), acute coronary syndrome (5%, n = 365), neoplasm (4%, n = 299) and chronic obstructive pulmonary disease (4%, n = 291). The most common principal or secondary chronic conditions recorded were diabetes (71%, n = 5,460), cancer (55%, n = 4,209) and arthropathies (32%, n = 2,469).

The ‘care type’ was ‘acute’ for 91% (n = 6,986) and ‘palliative’ for 9% (n = 683) of people. A large proportion of this cohort (43%, n = 3,279) had one or more palliative care encounters during a prior ED presentation or hospital admission in the 6 months prior. Of the 683 patients classified as palliative care-type this admission, the vast majority (n = 557, 82%) were not admitted from ED as palliative care-type, but rather were changed to this care type following admission.

## Discussion

This study establishes an incidence rate of older patients requiring imminent end-of-life care in the ED of ~6 per 1,000 presentations, increasing to 14 per 1,000 for people aged ≥90 years. As the population of older people grows in Australia and New Zealand, the number of people likely to need end-of-life care will concomitantly increase. In a larger institution with ~25,000 older persons presentations annually, ~3 individuals at imminent end of life per week would be expected. Evidence is limited in which to compare our incidence, beyond earlier research (for patients aged ≥60 years) that was 8.0 per 1,000 [14]. Future research is thus warranted to investigate more nuanced differences across age cohorts.

In our study, based on the palliative care encounter indicator, nearly half of older patients appeared to present with an understanding that they might be at their end of life. Based on other studies, a palliative approach would be appropriate for about one-quarter to one-half of older patients [15–17]. Despite guidelines and recommended geriatric ED model designs [30–32], few EDs are set up to manage the special needs of the older person [33, 34], regardless of whether they present at end of life. For example, in the United States only ⁓400 of >4,000 EDs are accredited geriatric EDs [35, 36]. Customisations of the ED for the older person include institution of geriatric-specific screening, implementation of ‘geriatric-friendly’ model(s) of care and deployment of equipment that minimises risk to older persons, such as large analogue clocks, raised toilet seats, beds that reduce the incidence of pressure areas and prevent falls, or single rooms for patients imminently dying [37]. Furthermore, in many countries, including Australia and New Zealand, the current health care architecture itself is not designed to manage the care needs of older people. Some older people approaching end of life can be cared for in different settings (at home or in their residential aged care facility), if adequate supports existed [38–43]. Innovation in ED and health system design to better support end-of-life care is urgently required [38, 39].

Although a prior palliative care encounter occurred for ~4 in 10 admitted patients, <1 in 10 had their care-type recorded as ‘palliative’, and most of this latter group left ED with an acute, not end-of-life, care-type. This suggests that it may be difficult for clinicians to recognise and/or acknowledge and/or declare imminent death, even for patients previously classified as receiving palliative care. However, this may also reflect clinical documentation not meeting certain criteria for ‘palliative care encounter’ coding [44].

In the ED, clinicians need to make quick decisions as to what kind of treatment to initiate for a patient that they just met and may only have limited information about. The decision as to whether it is appropriate to start and provide end-of-life care in their current ED visit may therefore be challenging [27], but benefits from shared decision-making [45]. A priori knowledge of patients’ and families’ wishes through established mechanisms such as Advanced Care Directives [46] or Voluntary Assisted Dying pathways can aid a rapid and comfortable decision for ED physicians, enabling a clear disposition or fast-tracked admission of the patient under their inpatient specialist care. To facilitate clinicians’ decision-making, we support movement towards national electronic advanced care planning registers; all individuals can upload their own advanced care directives or opt-in through their primary care physician to document their current wishes about the level of care to provide—whether all treatment measures or comfort-care focused measures, or anything in between. This information could be updated on my HealthRecord (in Australia) or another electronic platform that is accessible to the individual and his/her delegate(s), pre-hospital and hospital clinicians, as well as general practitioners and residential aged care facilities.

Our study identifies some risk factors for older people dying in ED rather than dying elsewhere, where patient preferences, spaces and resources may be better suited. These factors are summarised in Box 1. One or more of these risk factors may prompt ED clinicians to ask the ED ‘surprise’ question*, ‘Would I be surprised if my patient died this presentation?’* [47]*.*

**Box 1. Factors associated with dying in the emergency department vs elsewhere in-hospital** 

- Younger older adults (65–74 years)
- Arriving by ambulance
- Triage category of 1, 2 or 5
- Large or principal referral facilities
- Presenting with a circulatory system disorder
- Dying between noon and midnight
- Not identifying as an Aboriginal or Torres Strait Islander person

Amongst these factors, presentation between the hours of 12:00 and 17:59 conferred a decreased risk of dying in ED, whilst those that died in ED were more likely to die in the evening/night (between noon and midnight). We are unable to differentiate whether these findings could be because more acute presentations occurred at this time or due to resource restraints after-hours. Different health systems may have different after-hours bed management strategies; therefore, sites should assess this phenomenon at their facility and, as necessary, ascertain any systems issues that may impact admission processes after-hours. For example, access block after-hours may be due to decreased ability to accept new admissions without specialist review, insufficient ward staffing levels to provide end-of-life care or limited capacity for senior decision-making. One US study reports lack of 24-hour access to a palliative care team as one of the most important barriers to the provision of palliative care [48]. Recent evidence that overnight stays in ED involve risks for older people would advocate that older people be prioritised for admission to prevent harm [23]. We recommend emergency care and inpatient areas continue efforts towards 24-hour facilitation of people at end of life, as real-time palliative care and social work support for this population are needed [47]. We also recommend raising awareness of emergency medicine College policies that provide guidance in caring for older people [30] and people requiring end-of-life or palliative care [49] in the ED.

Older people who were Aboriginal and/or Torres Strait Islander were also less likely to die in ED in our study. Other studies have found a greater likelihood to die in a private residence for Māori people [50] and a preference to die ‘on country’, surrounded by family and friends for Aboriginal and/or Torres Strait Islander people [51]. Our finding suggests that these preferences may have been translated somewhat into the care received in this cohort’s EDs.

A triage category of 1, 2 or 5 was associated with an increased chance of dying in ED (compared to ATS 3). Older persons that present to ED and die shortly thereafter were triaged more frequently into the most acute categories (ATS 1 and 2) compared to the general population of Australia (73% this cohort versus 16% Australia-wide [7], respectively), an indicator that most of this critical cohort were appropriately identified as such. However, some patients (~1 in 4) were still triaged as 3, 4 or 5. This proportion is consistent with another Australian study [17]. This suggests, again, that imminent death is not easily recognisable for many older people. The finding that ATS 5 was associated with an increased risk of dying in ED may be an artefact due to the small numbers in this group, but also raises the concern that under-triage in older adult populations may occur beyond the well-known literature describing under-triage in trauma care of older people [52, 53]. Whilst current Australasian triage guidelines contain mention of ‘specific convention’ for older people in the ED that recognises the need to account for certain complexities [29], further research into the appropriateness of triage systems and sensitivity of triage practices as applied to older people is warranted.

## Limitations

This study uses administrative data collated by an independent not-for-profit organisation. Although >100 institutions contribute data to Health Roundtable, our findings may not be reflective of or generalisable to all EDs/hospitals in Australia, New Zealand or other countries. Data used in our study are extensive in terms of breadth but limited in terms of depth. Thus, we did not have information regarding baseline functionality, patient wishes and other comorbidities that may explain some of our findings.

Our use of routinely collected administrative health data for research purposes relies on coding practices, which may depend on clinical documentation, and can vary from institution to institution. Even though the assignment of diagnosis codes in EDs has been shown to have high agreement and reliability, variation of coding quality across hospitals can occur [54]. Furthermore, some variability between administrative coding and clinical documentation can exist, including with palliative care coding, due to medical record documentation not meeting criterial for coding [44]. Thus, our analysis pertaining to the use of the palliative care ICD-10 (Z51.5) code may under-represent the extent of palliative care received at end of life.

Our sample comprised patients who died within 48 hours of ED presentation. We did not have access to linked data from our study to death records and may therefore have missed some patients who died (post-ED discharge), potentially underestimating the incidence of the need for ED end-of-life care in this cohort.

## Conclusions

Our paper focuses on the older person dying within 48 hours, establishing an incidence rate of 6.43 per 1,000 older people in need of imminent end-of-life care in Australian and New Zealand EDs. Recommendations from this study include the following: (i) health care architecture within the ED and the community needs to evolve as about half of this older population was recognised to have palliative care needs prior to this presentation; (ii) the identification of imminent death is often difficult—we support the establishment of national advanced care planning registers to expedite appropriate care and facilitate admission processes; and (iii) to improve quality of care, implement regular audit systems for older people triaged into low-urgency categories (4 or 5) who die within 48 hours—people dying cannot wait. Consideration of the suitability of the triage system for older people should be a research priority.

## Data Availability

We are unable to share or make publicly available data used for this study due to proprietary and privacy requirements.
